# Sex-specific association of metabolic risk factors with brain ischemic lesions by severity and location

**DOI:** 10.1186/s13293-019-0254-6

**Published:** 2019-08-22

**Authors:** Hailuan Zeng, Weibin Shi, Wenhai Jiang, Shengxiang Rao, Beijian Huang, Hongmei Yan, Xin Gao

**Affiliations:** 1 Department of Endocrinology and Metabolism, Zhongshan Hospital, Fudan University, Shanghai, China; 2 Fudan Institute for Metabolic Diseases, Shanghai, China; 3 Medical Examination Center, Zhongshan Hospital, Fudan University, Shanghai, China; 4 Computer Network Center, Zhongshan Hospital, Fudan University, Shanghai, China; 5 Department of Radiology, Zhongshan Hospital, Fudan University, Shanghai, China; 6 Department of Ultrasonography, Zhongshan Hospital, Fudan University, Shanghai, China

**Keywords:** Sex-specific, Metabolic risk factors, Brain ischemic lesions, Severity, Location

## Abstract

**Background:**

Males and females exhibit significant differences in metabolism and in brain ischemic stroke and different features of brain ischemic lesions are related to different health outcomes. It is critical to understand sex differences in their associations to optimize prevention and intervention for both sexes. We aimed to investigate the sex-specific association of metabolic risk factors with brain ischemic lesions by severity and location.

**Methods:**

Five thousand seven hundred ninety-one participants who underwent comprehensive health examinations between Jan. 1, 2017, and Dec. 31, 2017, were enrolled. Clinical and laboratory data about metabolic risk factors were obtained. Brain ischemic lesions were further categorized by severity (mild ischemic lesions or severe infarct lesions) and location (strictly lobar or deep brain/infratentorial areas) based on brain magnetic resonance imaging reports. Sex- and age-specific detected rates were calculated, and generalized linear models and multinomial logistic regression were used to analyze the associations between metabolic risk factors and the presence, severity, and location of ischemic lesions stratified by sex.

**Results:**

A total of 2712 (46.8%) participants had at least one brain ischemic lesions. Age (adjusted OR, 1.10 [1.10–1.11], *p* < 0.001) and hypertension (adjusted OR, 1.42 [1.22–1.64], *p* < 0.001) were generally associated with higher risks of brain ischemia in both sexes. Metabolic syndrome was associated with greater adjusted ORs for brain ischemia with different severity and location in men (adjusted ORs between 1.23 and 1.49) but not in women. Overweight and obesity were related to lesions located strictly in lobar in men (adjusted OR, 1.23 and 1.33, respectively) and lesions located in deep brain/infratentorial areas in women (adjusted OR, 1.57 and 2.26, respectively).

**Conclusions:**

Metabolic syndrome was associated with brain ischemic lesions in men but not in women. Higher body mass index was related to ischemic lesions located in lobar in men and in deep brain/infratentorial areas in women. Its mechanisms remain to be further investigated.

## Background

Brain ischemic lesions are frequently seen on brain magnetic resonance imaging (MRI) especially in the elderly population and have been reported to be associated with cognitive impairment [1], depression [2], stroke [3], mortality [4], and other adverse health outcomes [5]. Although the incidence of total stroke was greater among males, females have a higher incidence of ischemic stroke and suffer worse outcomes relating to the severity of deficits and disability from stroke, yet they are more likely to survive [6–8]. There are many potential confounding factors, and more detailed research is needed to shed light on this complex relationship.

Findings in recent years suggest that not only the presence, but also the distribution, multiplicity, size, and side of brain ischemic lesions are related to different health outcomes and showed sex disparity in new infarct risk and cognitive decline [4, 9–12]. For example, results from the Chicago Health and Aging Project (CHAP) indicated that cortical, multiple, large, and bilateral infarcts were associated with lower cognition, particularly worse memory and perceptual speed [11]. All these results suggest that the features of brain ischemic lesions with sex differences might be indicative of different etiologies and pathogenic mechanisms that require investigations.

Risk factors for silent brain infarcts (SBI) have been extensively explored and well documented [5, 13–15]. Metabolic syndrome was reported to be strongly associated with risks of SBI and the adjusted odds ratio (OR) increased with the number of its components [16]. While advanced age and hypertension are well known to be associated with SBI as well as clinically defined ischemic stroke[15, 17–19], the relationships between brain infarctions and another single component of metabolic syndrome such as impaired fasting glucose and dyslipidemia remain controversial [16, 19–22]. Besides, fatty liver was recently pointed out to be an emerging predictor of stroke risk, severity, and outcome [23], yet there is limited information from large imaging studies on its association with brain ischemic lesions. Sex differences exist in nearly all of the components of metabolic syndrome, for example, males are more likely to have fat distribution in visceral areas while females in subcutaneous depots [24], females tend to have greater insulin sensitivity although they have greater percentage of fat mass than males, premenopausal females generally have higher HDL levels while males have higher plasma triglyceride levels, but lipoprotein profiles become more similar to males in postmenopausal females [25]. In this regard, we hypothesized that older age and hypertension were risk factors for brain ischemia in both sexes, while sex differences in associations between brain ischemic lesions and metabolic syndrome or its components may exist, especially taking lesion features such as severity and location into account. And we explored our hypothesis in a large cross-sectional health examination data set.

## Methods

### Study population

Every year, about 100,000 people visit our Medical Examination Center in Zhongshan Hospital, Fudan University (Shanghai, China), for health examinations. Some of them were told by doctors in local community hospitals that they were at high risk of some certain diseases (e.g., cerebrovascular diseases) or are concerned about their health status and most of them are free of symptoms. From January to December in 2017, 13,007 subjects received a comprehensive health examination. After a preliminary consultation on demographic characteristics and a basic physical examination by trained physicians, they voluntarily undergo a series of blood tests, urine tests, and imaging examinations to screen for organ dysfunction, vascular disease, cancer, and other disorders. Their mean age was 47.9 (SD 11.3) years and 55.2% were men. About half of the subjects underwent brain MRI scan (*n* = 5860, 45.1%). After excluding 6 subjects under 20 years old and 63 without complete data, 5791 subjects were enrolled in this study. The study was approved by the human research ethics committee of Zhongshan hospital, Fudan University, with a waiver of consent because this study did not involve contact with participants or any intervention.

### Clinical and laboratory data

All subjects underwent comprehensive medical examinations by trained personnel with standardized instruments. Demographic and laboratory findings including age, sex, height, weight, blood pressure, fasting glucose, hemoglobin A1C (HbA1c), total cholesterol (TC), high-density lipoprotein cholesterol (HDL-C), low-density lipoprotein cholesterol (LDL-C), triglycerides (TG), lipoprotein a (Lp(a)), aspartate aminotransferase (AST), alanine transaminase (ALT), gamma-glutamyl transferase (GGT), uric acid (UA), blood urea nitrogen (BUN), serum creatinine (Scr) were obtained and analyzed. All laboratory determinations were carried out using standard laboratory methods. For participants who had undergone more than one health checkup, data from the earliest examination were used.

### Liver ultrasonic examination

Hepatic ultrasonographies (Vivid 7, GE Healthcare, Horten, Norway) were carried out by experienced sonologists. The diagnosis of fatty liver was made on the basis of characteristic ultrasonographic features consistent with dense and enhanced echogenicity of the liver parenchyma with obvious posterior attenuation of the echo, vessel blurring, and narrowing of the lumen of the hepatic veins [26].

### Brain MRI and ischemic lesions

A 3.0-Tesla MRI scanner (GE Healthcare, Milwaukee, WI) was used to obtain T1-weighted, T2-weighted, fluid-attenuated inversion recovery (FLAIR), and diffusion-weighted imaging (DWI) sequences. After the MRI scan, formal reading reports containing image findings and diagnosis were given by two individual radiologists from the Department of Radiology in 2 days, disagreements were settled after discussion with a third board-certified radiologist. All of them were blinded to the subjects’ clinical information and were unaware of the project goals. In brief, brain ischemic lesions were recognized as focal high-intensity areas identified on a T2-weighted image coinciding with low-intensity areas on a T1-weighted image, and ischemic/infarct ones with single/multiple locations were recorded. All kinds of brain ischemic lesions seen on MRI images were reported regardless of their size, whether they were symptomatic or silent, old or new. Screen results were extracted from the medical databases stored in the computer network center in our hospital. For researching convenience, brain ischemic lesions were categorized into mild ischemic or severe infarct lesions and into one of three locations: lobar (cortical gray and subcortical white matter of periventricular, centrum ovale, and corona radiate), deep brain (gray matter of basal ganglia and thalamus, and the white matter of corpus callosum, internal and external capsule), and infratentorial (brainstem and cerebellum) according to MRI reports. No distinction was made between participants with the size and number of ischemic lesions on their scan.

### Metabolic status definitions

Hypertension was defined by a systolic blood pressure (SBP) ≥ 140 mmHg or a diastolic blood pressure (DBP) ≥ 90 mmHg. Dyslipidemia was defined as increased total cholesterol ≥ 6.2 mmol/L, LDL-C ≥ 4.1 mmol/L, triglyceride levels ≥ 2.3 mmol/L, or decreased HDL-C < 1.0 mmol/L]) [27]. Diabetes mellitus was defined as fasting plasma glucose ≥7.0 mmol/L or HbA1c ≥ 6.5%, and pre-diabetes was defined as fasting plasma glucose between 5.6 mmol/L and 6.9 mmol/L or HbA1c between 5.7% and 6.4%. Fatty liver was categorized into mild to moderate fatty liver and severe fatty liver according to ultrasonic examination results. BMI was calculated from measured weight and height and categorized as follows: normal (BMI < 24 kg/m^2^), overweight (24 kg/m^2^ ≤ BMI < 28 kg/m^2^), and obese (BMI ≥ 28 kg/m^2^). Subjects were labeled as having metabolic syndrome by the presence of three or more of the following: (1) obesity with a BMI ≥ 25 kg/m^2^, (2) blood pressure ≥ 130/85 mmHg, (3) fasting glucose ≥ 100 mg/dL (5.6 mmol/L), (4) triglyceride ≥ 150 mg/dL (1.7 mmol/L), and (5) high-density lipoprotein cholesterol < 40 mg/dL (1.03 mmol/L) in men and < 50 mg/dL (1.3 mmol/L) in women [28].

### Statistical analysis

All statistical analyses were performed using R, version 3.4.4 (R Programming). Continuous variables are expressed as mean (SD) or median [IQR], and categorical data are expressed as count (percentage). Student’s *t* test or the nonparametric Mann-Whitney *U* test was used for intergroup comparisons of continuous data whereas the chi-squared test was used for comparisons of categorical variables. The prevalence and characteristics of ischemic lesions on MRI were calculated in 10-year age strata.

At first, univariate generalized linear models were made to analyze the associations of age, sex, and metabolic risk factors with the presence of brain ischemic lesions followed by models adjusted for age and sex (age and sex were adjusted for each other), and a full model containing all covariates except for metabolic syndrome (to avoid collinearity) was applied. After that, generalized linear models and multinomial logistic regressions adjusted for all covariates except for sex were made stratified by sex to analyze the associations between metabolic status (except for metabolic syndrome, which results were adjusted for age only) and presence, severity (none, ischemic, infarct), and location (strictly lobar, deep brain, or infratentorial with or without lesions in lobar) of lesions. All statistical tests were two-tailed, and *p* < 0.05 was considered statistically significant.

## Results

### Participant characteristics

**Fig. 1:**
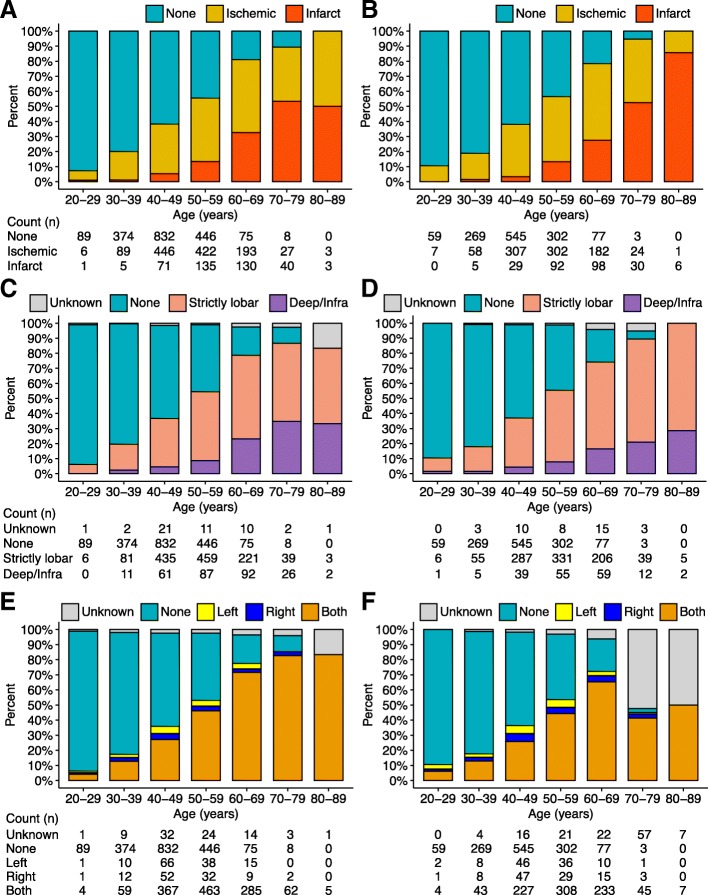
Detection results of brain ischemic lesions stratified by age and sex. **a**, **b** Detected rate of brain ischemic and infarct lesion in male (**a**) and female (**b**) stratified by age. **c**, **d** Location of brain ischemic/infarct lesion in male (**c**) and female (**d**) stratified by age. **e**, **f** Side of cerebral hemisphere of brain ischemic/infarct lesion in male (**e**) and female (**f**) stratified by age

A total of 5791 participants were included in the analysis. The mean (SD) age of the population was 48.8 (10.1) years with a range of 20–88 years, and 3395 (58.6%) were men. Brain ischemic/infarct lesions were detected in 2712 (46.8%) participants. Compared to females, males have higher BMI and higher incidence of metabolic syndrome including all its components (Table 1). The detected rates of ischemic lesions by severity and location stratified by sex and age groups are presented in Fig. 1. Sex distribution was balanced among age groups. In both sexes, the detected rates of brain ischemic/infarct lesions increased dramatically with age. Older women tended to have severe infarct lesions while lesions located in deep or infratentorial areas were more prevalent in older men (Fig. 1).

**Table 1:** Baseline characteristics of the study population

	Overall (*n* = 5791)	Female (*n* = 2396)	Male (*n* = 3395)	*p* value
Age, year	48.8 (10.1)	49.2 (10.4)	48.6 (9.92)	0.024
BMI, kg/m^2^	24.0 (3.34)	22.7 (3.12)	25.0 (3.19)	< 0.001
SBP, mmHg	116 (14.7)	114 (15.6)	117 (13.9)	< 0.001
DBP, mmHg	78.6 (9.5)	76.1 (9.3)	80.4 (9.2)	< 0.001
Fasting glucose, mmol/L	5.16 (1.19)	5.02 (0.97)	5.26 (1.32)	< 0.001
HbA1C, %	5.54 (0.74)	5.45 (0.62)	5.61 (0.82)	< 0.001
TG, mmol/L	1.77 (1.38)	1.35 (0.96)	2.07 (1.55)	< 0.001
TC, mmol/L	5.06 (0.99)	5.02 (0.96)	5.09 (1.01)	0.004
HDL-C, mmol/L	1.33 (0.373)	1.52 (0.376)	1.20 (0.306)	< 0.001
N-HDL-C, mmol/L	3.73 (1.01)	3.50 (0.96)	3.89 (1.02)	< 0.001
LDL-C, mmol/L	2.96 (0.875)	2.90 (0.862)	3.01 (0.882)	< 0.001
Lp(a), mg/L	114 [53.3, 234]	130 [67.0, 253]	103 [46.0, 219]	< 0.001
APOB, g/L	0.923 (0.224)	0.870 (0.216)	0.960 (0.222)	< 0.001
APOA-1, g/L	1.52 (0.269)	1.62 (0.268)	1.45 (0.247)	< 0.001
APOE, mg/L	45.7 (16.7)	44.3 (14.3)	46.6 (18.2)	0.002
ALT, U/L	20.0 [13.5, 31.1]	14.5 [10.8, 20.9]	25.1 [17.6, 37.5]	< 0.001
AST, U/L	21.0 [17.4, 26.0]	19.0 [16.0, 23.0]	22.5 [19.0, 28.0]	< 0.001
GGT, U/L	27.0 [17.0, 48.0]	17.3 [13.0, 24.6]	38.0 [25.0, 63.0]	< 0.001
UA, umol/L	339 (90.0)	273 (61.3)	386 (77.1)	< 0.001
BUN, mmol/L	4.93 (1.20)	4.65 (1.20)	5.13 (1.17)	< 0.001
Scr, umol/L	76.0 (16.3)	62.2 (9.43)	85.8 (12.7)	< 0.001
CRP, mg/L	0.99 [0.54, 1.89]	0.79 [0.45, 1.50]	1.12 [0.63, 2.18]	0.000
Hypertension, *n* (%)	1205 (20.8%)	383 (16.0%)	822 (24.2%)	< 0.001
Dyslipidemia, *n* (%)	2155 (37.2%)	521 (21.7%)	1634 (48.1%)	< 0.001
Pre-diabetes, *n* (%)	1483 (25.6%)	567 (23.7%)	916 (27.0%)	< 0.001
Diabetes, *n* (%)	299 (5.2%)	59 (2.5%)	240 (7.1%)	< 0.001
Mild to moderate fatty liver, *n* (%)	1275 (22.0%)	345 (14.4%)	930 (27.4%)	< 0.001
Severe fatty liver, *n* (%)	1200 (20.7%)	297 (12.4%)	903 (26.6%)	< 0.001
Overweight, *n* (%)	2194 (37.9%)	605 (25.3%)	1589 (46.8%)	< 0.001
Obese, *n* (%)	657 (11.3%)	139 (5.8%)	518 (15.3%)	< 0.001
Metabolic Syndrome, *n* (%)	1407 (24.3%)	358 (14.9%)	1049 (30.9%)	< 0.001

Data presented as mean (SD) or median (IQR) and *n* (%)

*BMI* body mass index, *SBP* systolic blood pressure, *DBP* diastolic blood pressure, *TG* triglyceride, *TC* total cholesterol, *HDL-C* high-density lipoprotein cholesterol, *N-HDL-C* non-high-density lipoprotein cholesterol, *LDL-C* low-density lipoprotein cholesterol, *Lp(a)* lipoprotein a, *ALT* alanine aminotransferase, *AST* aspartate aminotransferase, *GGT* gamma-glutamyl transferase, *UA* uric acid, *BUN* blood urea nitrogen, *Scr* serum creatinine, *CRP* C-reactive protein

### Association of metabolic status with the risk of brain ischemic lesions

In univariate generalized linear models, older age, hypertension, pre-diabetes, diabetes, overweight, obesity, fatty liver, and metabolic syndrome were strongly associated with the risk of harboring brain ischemic lesions on MRI. The ORs ranged between age 1.10 (95% CI, 1.10–1.11) and diabetes 1.92 (95% CI, 1.51–2.44), while the associations between the risk of harboring brain ischemic lesions and sex or dyslipidemia were of no significance (Table 2). After adjusting for age and sex, age (adjusted for sex), hypertension, overweight, obesity, and metabolic syndrome were still significant (Table 2). In a multiple analysis adjusted for all covariates, the difference was significant only for age and hypertension with adjusted ORs of 1.10 (95% CI, 1.10–1.11) and 1.42 (95% CI, 1.22–1.64), respectively (Table 2).

**Table 2:** Relationships between metabolic status and the risk of harboring brain ischemic lesions

Variables	Unadjusted	*p* value	Adjusted for age and sex		Multiple	*p* value
	OR (95% CI)		OR (95% CI)	*p* value	OR (95% CI)	
Age	1.10 (1.10–1.11)	< 0.001	1.10 (1.10–1.11)	< 0.001	1.10 (1.10–1.11)	< 0.001
Male	0.95 (0.85–1.05)	0.312	1.00 (0.89–1.12)	0.974	0.94 (0.83–1.06)	0.308
Hypertension	1.91 (1.68–2.17)	< 0.001	1.42 (1.23–1.64)	< 0.001	1.42 (1.22–1.64)	< 0.001
Dyslipidemia	1.06 (0.96–1.18)	0.257	1.04 (0.92–1.17)	0.559	1.02 (0.90–1.16)	0.720
Pre-diabetes	1.67 (1.48–1.88)	< 0.001	0.90 (0.79–1.03)	0.143	0.86 (0.75–0.99)	0.041
Diabetes	1.92 (1.51–2.44)	< 0.001	0.94 (0.72–1.23)	0.658	0.90 (0.69–1.18)	0.453
Overweight	1.31 (1.18–1.47)	< 0.001	1.14 (1.00–1.29)	0.047	1.13 (0.99–1.30)	0.074
Obesity	1.28 (1.08–1.51)	0.005	1.23 (1.01–1.49)	0.035	1.24 (1.00–1.54)	0.052
Mild to moderate fatty liver	1.30 (1.15–1.49)	< 0.001	1.14 (0.99–1.32)	0.074	1.06 (0.91–1.24)	0.475
Severe fatty liver	1.15 (1.01–1.31)	0.042	1.00 (0.86–1.16)	0.974	0.89 (0.74–1.06)	0.181
Metabolic Syndrome	1.46 (1.29–1.65)	< 0.001	1.20 (1.05–1.38)	0.007	–	–

Values represent estimated odds ratios with 95% confidence interval for any brain ischemic lesions with reference to normal status

### Brain ischemic severity and metabolic status

**Fig. 2:**
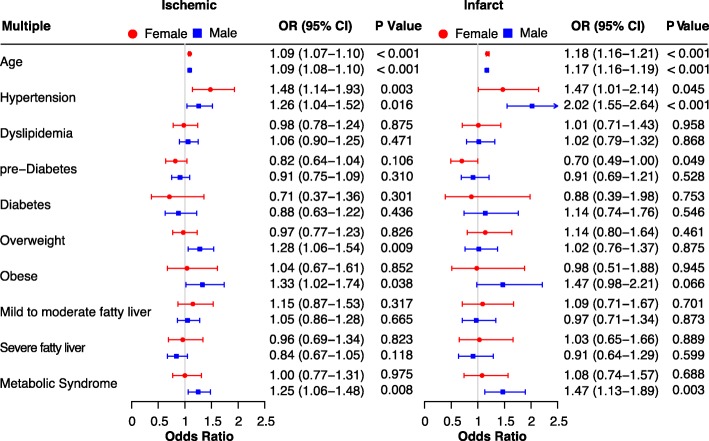
Multinomial log regression examining the relationship between metabolic status and the risk of harboring ischemic or infarct lesions

Multinomial regression adjusted for all covariates showed that older age was strongly associated with the risk of brain ischemic lesions and infarct lesions and the adjusted ORs were almost the same in both sexes: 1.09 for ischemic lesions and 1.18 for infarct lesions. Hypertensive status was associated with an increased risk of harboring ischemic lesions in both female (adjusted OR, 1.48; 95% CI, 1.14–1.93) and male (adjusted OR, 1.26; 95% CI, 1.04–1.52), as well as an increased risk of harboring infarct lesions (female adjusted OR, 1.47; 95% CI, 1.01–2.14 and male adjusted OR, 2.02; 95% CI, 1.55–2.64). Overweight and obesity were significantly associated with a higher risk of harboring ischemic lesions but not infarct lesions in male (adjusted OR, 1.28 and 1.33 vs adjusted OR, 1.02 and 1.47), whereas those in female were not significant. Dyslipidemia, pre-diabetes, diabetes, and fatty liver were not significantly associated with harboring either brain ischemic lesions or infarct lesions in both sexes. A status of metabolic syndrome was found to have a harmful effect in male (adjusted ORs for ischemia and infarction were 1.25 [95% CI, 1.06–1.48; *p* value, 0.008] and 1.06 [95% CI, 1.13–1.89; *p* value, 0.003], respectively) but not in female (Fig. 2).

### Brain ischemic lesion location and metabolic status

**Fig. 3:**
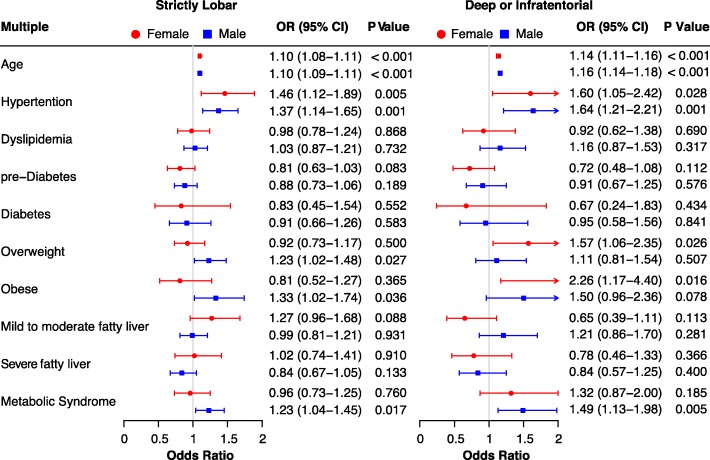
Multinomial log regression examining the relationship between metabolic status and the location of brain ischemic lesions

The effect of metabolic status on brain ischemic lesion locations was also investigated with multinomial logistic regression adjusted for all covariates. Again, age increasement and hypertension were associated with higher ORs for lesions located both strictly in lobar and deep or infratentorial areas in both sexes. Additionally, overweight and obesity were associated with significantly greater ORs for lesions located strictly in lobar in male (adjusted OR, 1.23; 95% CI, 1.02–1.48 and adjusted OR, 1.33; 95% CI, 1.02–1.74, respectively) and lesions located in deep brain or infratentorial areas in female (adjusted OR, 1.57; 95% CI, 1.06–2.35 and adjusted OR, 2.26; 95% CI, 1.17–4.40, respectively). After adjusting for age, metabolic syndrome had a higher adjusted OR for lesions located strictly in lobar (adjusted OR, 1.23; 95% CI, 1.04–1.45; *p* value, 0.017) and in deep brain or infratentorial areas (adjusted OR, 1.49; 95% CI, 1.13–1.98; *p* value, 0.005) in male but showed no significance in female (Fig. 3).

## Discussion

In a population of 5791 Chinese undergoing medical examinations, a total of 2712 (46.8%) participants had at least one brain ischemic lesion. The detected rate increased steadily with age and was balanced in males and females. The prevalence of hypertension, pre-diabetes, diabetes, fatty liver, overweight, and metabolic syndrome was higher in males. Older age and hypertension were major risk factors for brain ischemic lesions in both sexes while metabolic syndrome showed significantly greater adjusted ORs in men but not in women. Overweight and obesity were related to lesions located strictly in lobar in men and lesions located in deep brain or infratentorial areas in women.

The prevalence of brain ischemic/infarct lesions in our study was relatively high compared with previous works on SBI [13, 15, 29–32]. There are two possible reasons for these inconsistent findings: one is that unlike healthy participants receiving health checkups paid by their employers in some other studies, the majority of the participants who visited our examination center and asked for a brain MRI scan were concerned about their worse health status such as diabetes, obesity, and other comorbidities; the other is that we reported all kinds of brain ischemic lesions seen on MRI images regardless of their size, whether they were symptomatic or silent, old or new, while others focusing on SBI included only lesions with the size of 3–15 mm and were silent ones and subjects with stroke, transient ischemic attack, or other cardiovascular diseases (and were more likely to have brain infarctions) were excluded.

As expected, older age and hypertension were main risk factors for brain ischemic lesions regardless of the severity and location, which was in accordance with similar studies [19, 29–31]. Studies from Korea and Japan reported adjusted ORs of 1.06–1.09, 1.54–3.75, and 1.68–2.43 for the associations between silent brain infarctions and age, elevated blood pressure, and metabolic syndrome, respectively [16, 19, 21]. Similarly, in our study, age, hypertension, and metabolic syndrome showed significance in multinomial regression models with adjusted ORs of 1.10 (95% CI, 1.10–1.11), 1.42 (95% CI, 1.22–1.64), and 1.20 (95% CI, 1.05–1.38), respectively. The associations between dyslipidemia, pre-diabetes, diabetes, overweight, obesity, and fatty liver and the presence of brain ischemic or infarct lesions in the general population showed significantly higher unadjusted ORs but were insignificant after adjustment, which might indicate that they might not be independent risk factors for brain ischemia.

Metabolic syndrome was a risk factor for brain ischemia in men but not in women after adjusting for age. This was in accordance with a few studies indicated no increased risk of metabolic syndrome-related ischemic stroke especially in women [33, 34], although some showed that both males and females with metabolic syndrome are at higher risks for stroke [35]. The inconsistent results may be explained by different study designs and definitions and considering different variables for adjustment. It is surprising that little is known about the sex differences in the relationships between metabolic syndrome and silent brain ischemia or infarction. In our study, most of the participants with brain ischemic lesions were at an early stage with no symptoms, and our results suggested that metabolic syndrome might play a role in brain ischemia before the onset of stroke in men. It is noteworthy that male participants were at worse metabolic status (Table 1), but the detected rates of lesions were balanced in both sexes (Fig. 1), which implied that brain ischemia in females was more influenced by age, especially in those older than 50 years.

Another interesting finding was the disparate impacts of overweight and obesity (calculated from BMI) on lesion severity and location in different sexes. Higher BMIs were associated with ischemic lesions but not infarct ones in male participants, which might give a hint that it was a trigger factor for brain ischemia but would not lead to advanced infarctions. Surprisingly, higher BMIs were significantly related to a higher risk of lesions located strictly in lobar in men and lesions located in deep brain or infratentorial regions in women. Some publications have addressed the relationship between BMI and intracranial location of the brain hemorrhage [36], but there was a lack of information on the sex-specific associations between BMI and location of brain ischemia. A prospective case-control study in Spain reported that increased BMI was related to a lower risk of stroke in men and abdominal obesity was associated with ischemic stroke in women [37]. Unfortunately, data about abdominal obesity including waist circumference and waist-to-height ratio of the participants were not obtained in the present study, but our results emphasized the need to uncover sex differences to better understand the underlying mechanisms. Sex-related differences in fat distribution and its impacts on cardiovascular diseases are well established, and thus it would be interesting to further investigate whether sex-related disparity of fat distribution is associated with specific locations of brain ischemia and ischemic stroke.

The strength of our study included the large number of participants representing a broad range of age (20–88 years old), the detailed analysis of brain ischemic lesions including the presence, severity, and location, and the use of age- and sex-stratified metabolic status. Our study had several limitations. First, this was a secondary analysis of medical examination data, and thus the size, multiplicity, shape, and location of the brain lesions could not be reviewed and scored, yet the reports were given by experienced radiologists and checked by clinicians in our hospital who were blinded to the study. Second, we did not exclude participants who had taken medications, which might have underestimated the associations of brain ischemic lesions with hypertension, diabetes, or dyslipidemia. Third, information on smoking status, drinking status, physical activities, and some other comorbidities such as atrial fibrillation [38, 39], carotid artery stenosis, and renal dysfunction [40] of the participants were not available and might be confounders or mediators. Another limitation to our study is the observational design and the inability to observe causal effects.

## Conclusions

In summary, this study found that older age and hypertension were generally associated with an elevated risk of brain ischemia in both sexes while metabolic syndrome was related to higher risk in men but not in women. Higher BMI was associated with ischemic lesions located strictly in lobar in men and lesions located in deep brain or infratentorial regions in women. Sex-related differences in associations of obesity with brain ischemic areas need further investigations to better understand the underlying pathological processes and mechanisms.

## Data Availability

The datasets generated and/or analyzed during the current study are available from the corresponding author on reasonable request.
